# Fibroblast‐derived neuropilin 1 alleviates renal fibrosis progression

**DOI:** 10.1002/path.70009

**Published:** 2025-12-23

**Authors:** Yunzhu Shen, Sandrine Placier, Liliane Louedec, Perrine Frère, Sophie Vandermeersch, Stefanny Figueroa, Hélène François, Christos E Chadjichristos, Camille Cohen, Christos Chatziantoniou, Amélie Calmont

**Affiliations:** ^1^ INSERM U1155, Hôpital Tenon Sorbonne Université Paris France; ^2^ Service de Néphrologie Hémodialyse, AP‐HP, Université Paris Cité, Centre de recherche sur l'inflammation, INSERM U1149, Hôpital Bichat ‐ Claude Bernard Paris France; ^3^ INSERM U942 MASCOT Université Paris Cité Paris France

## Abstract

Chronic kidney disease (CKD) is a major global health challenge affecting over 10% of the adult population. A hallmark of CKD progression is the transdifferentiation of kidney fibroblasts into extracellular matrix‐producing myofibroblasts, a key mechanism involved in the decline of kidney function and the development of kidney failure. Fibroblasts maintain the structural integrity of the kidney and support epithelial survival, repair, and regeneration after acute kidney injury. Maladaptive repair is a failure to resolve fibroblast activation, which ultimately progresses to chronic injury and CKD. In this study, we showed that the membrane‐bound coreceptor neuropilin 1 (NRP1) was essential to maintain fibroblast function and prevent their transdifferentiation into myofibroblasts. We used the myelin protein zero‐Cre (*P0‐Cre*) to specifically abrogate *Nrp1* in kidney resident fibroblasts during fibrosis progression. We employed kidney‐induced interstitial fibrosis models combined with a lineage‐tracing strategy, single‐cell RNA sequencing analysis, and *ex vivo* explant cultures to reveal a cell autonomous protective role for NRP1 in limiting fibrosis. Furthermore, we extended the analysis by showing that *Nrp1* conditional mutants were more prone to develop cardiac fibrosis in a mouse model of heart failure. Collectively, these findings provide new insights into the signalling pathways controlling the transition from acute to chronic kidney disease conversion and identify NRP1 as a novel regulator of fibroblast supportive function. © 2025 The Author(s). *The Journal of Pathology* published by John Wiley & Sons Ltd on behalf of The Pathological Society of Great Britain and Ireland.

## Introduction

Chronic kidney disease (CKD) is a rapidly growing health problem and represents a serious threat in current medicine with a prevalence reaching >10% worldwide [1]. Regardless of the initial insult, CKD is characterised by progressive destruction of the renal parenchyma and loss of functional nephrons, the filtering units of the kidney. Renal fibrosis is the common endpoint of CKD, whose hallmark is deposition of pathological matrix by myofibroblasts [2]. Lineage‐tracing experiments, together with single‐cell RNA sequencing (scRNAseq) analysis, have demonstrated that renal resident fibroblasts are the principal producers of extracellular matrix and scar tissue in both humans and mice [3, 4]. In basal conditions, renal fibroblasts provide structural support for nephrons by synthesising essential proteins to organise the extracellular matrix (ECM) and maintain organ homeostasis. During acute kidney injury (AKI), early activation of fibroblasts is crucial and required for kidney repair and regeneration [5]. However, maladaptive repair following AKI contributes to CKD as kidney fibroblasts transdifferentiate into myofibroblasts and promote progression of organ fibrosis [6].

Neuropilin 1 (NRP1) is a conserved membrane‐bound coreceptor for class 3 semaphorins and specific isoforms of vascular endothelial growth factor A (VEGFA) [7]. NRP1 displays pleiotropic functions, including the regulation of vascular and neuronal development [8], cancer aggressiveness [9], and fibrosis progression [10, 11]. In the kidney, NRP1 is expressed in mesangial cells of the glomerulus and in juxtaglomerular cells [12, 13, 14], where it regulates glomerular mesangial cell recruitment [12], glomerular basement membrane composition [14], and renin production [13]. During CKD, upregulation of NRP1 in distal tubules promotes fibrogenesis by activating Smad3 signalling and ECM synthesis by myofibroblasts [11]. However, NRP1 is also expressed in kidney fibroblasts [13], where its function in CKD initiation and progression is unknown. In this study, we used a genetic approach to knockout *Nrp1* specifically in kidney fibroblasts and analysed the impact of *Nrp1* deletion in two well‐characterised models of CKD: unilateral ureteral obstruction (UUO) and folic acid (FA)‐induced nephrotoxicity.

## Materials and methods

### Ethics approval

Animal experiments were carried out according to the guidelines approved by the French Ethics Committee APAFIS No. 11991.

### Mouse strains

We used the *Nrp1* ^*flox/flox*^ (MGI:3512101) [8], *P0‐Cre* (C57BL/6J‐Tg(P0‐Cre)94Imeg, ID148, CARD Japan) [15], and *Rosa* ^*YFP*^ (MGI:2449038) [16] alleles to generate the appropriate animals for the experimental study. *Nrp1* conditional mutants (*Nrp1‐ko*: *P0‐Cre; Nrp1* ^*flox/flox*^; *Rosa* ^*YFP*^), WT littermate controls (*Nrp1* ^*flox/+*^) and WT controls (*P0‐Cre; Rosa* ^*YFP*^) were maintained on a C57Blk/6J genetic background. The numbers of mice and/or samples (*n*) are listed in the figure legends.

### Immunolabelling

For immunolabelling, kidneys were fixed in 4% paraformaldehyde (19208, Electron Microscopy Sciences, Hatfield, PA, USA) in PBS and processed as 7‐μm frozen or 4‐μm paraffin sections according to standard protocols. In brief, sections were permeabilised in 0.5% Triton X‐100 for 5 min and incubated in blocking buffer for 1 h at room temperature (PBS containing 10% BSA, 10% goat serum, and 0.1% Triton X‐100), followed by incubation with primary antibodies in blocking buffer at 4 °C overnight. For paraffin sections, heat‐induced antigen retrieval in Target Retrieval Solution Citrate pH 6 (Dako, Glostrup, Denmark) was performed after deparaffinisation and rehydration; sections were then blocked and processed as described earlier.

Primary antibodies included goat anti‐NRP1 (AF 566, R&D Systems, Minneapolis, MN, USA), rabbit anti‐GFP (A6455, Invitrogen, Waltham, MA, USA) Ki‐67 monoclonal antibody (14‐5698‐82, Invitrogen), mouse anti‐alpha‐smooth muscle actin (α‐SMA) (A2547, Sigma‐Aldrich, St. Louis, MO, USA), and rabbit anti‐fibroblast activation protein (ab207178, Abcam, Cambridge, UK). Secondary antibodies were Alexa Fluor‐conjugated antibodies (Life Technologies and Jackson ImmunoResearch, West Grove, PA, USA). Fluorescent images were acquired using an Olympus IX83 inverted microscope (Hachioji, Tokyo, Japan). For quantification, kidney sections were imaged using a Hamamatsu NanoZoomer 2.0‐HT slide scanner (Hamamatsu, Shizuoka, Japan) and quantified with QuPath (Edinburgh, UK) under blinded conditions [17]. Three sections per specimen were imaged and scanned, and values were averaged for each mouse. Paraffin sections (4 μm thick) were stained with Picrosirius red, and interstitial fibrosis was quantified using QuPath as previously described [13]. For Ki67 and GFP double staining, or α‐SMA and GFP double staining, two training images were generated to produce staining vectors. A script was then used to quantify double‐positive cells for Ki67 and GFP relative to DAPI counterstain or α‐SMA‐positive cells normalised to GFP.

### Kidney and heart chronic injury models

Experiments were performed using either male or female mice, age 8–12 weeks, randomly allocated to control or treated groups. FA (240 mg/kg) (F7876, Sigma‐Aldrich) in vehicle (0.3 m NaHCO_3_) (S5671, Sigma‐Aldrich) or vehicle only was administered by i.p. injection [18]. After 48 h, blood samples were collected and kidney function assessed by measuring blood urea and creatinine levels. Three weeks after injection, kidneys were collected to assay fibrosis progression in the chronic phase. UUO was performed as previously described [19], and kidneys were collected 10 days following surgery. For heart fibrogenesis, adult males received a constant infusion of angiotensin II (AngII) (2 mg/g/day; A2900, Sigma‐Aldrich) at a constant rate through an osmotic pump (ALZET, CA, USA) for 28 days.

### Single‐cell RNAseq data analysis

Publicly available scRNAseq data for 12 patients with kidney disease from Kuppe *et al* [4], including matrix count and annotations, were analysed as previously described [20] and downloaded from Zenodo data archive (https://doi.org/10.5281/zenodo.4059315). These data concern human kidney mesenchymal cells (pericytes and fibroblasts) from patients with or without CKD. Six clusters were identified as previously described [20]. *NRP1* expression was assessed using the FeaturePlot and VlnPlot functions in the Seurat Package (https://satijalab.org/seurat/).

### Statistical analyses

All data are presented as mean ± SD. Statistical significance was determined using Student's *t*‐test when two groups conformed to normal distribution, Mann–Whitney *U* test when two groups did not conform to normal distribution, and one‐way ANOVA when there were more than three groups followed by a Tukey's *post hoc* test. A *p* < 0.05 was considered statistically significant. Data were analysed using GraphPad Prism 8 (GraphPad Software, San Diego, CA, USA). IBM SPSS Statistics 25 (IBM, Armonk, NY, USA) was used to assess whether the data followed a normal distribution.

## Results

**Figure 1:**
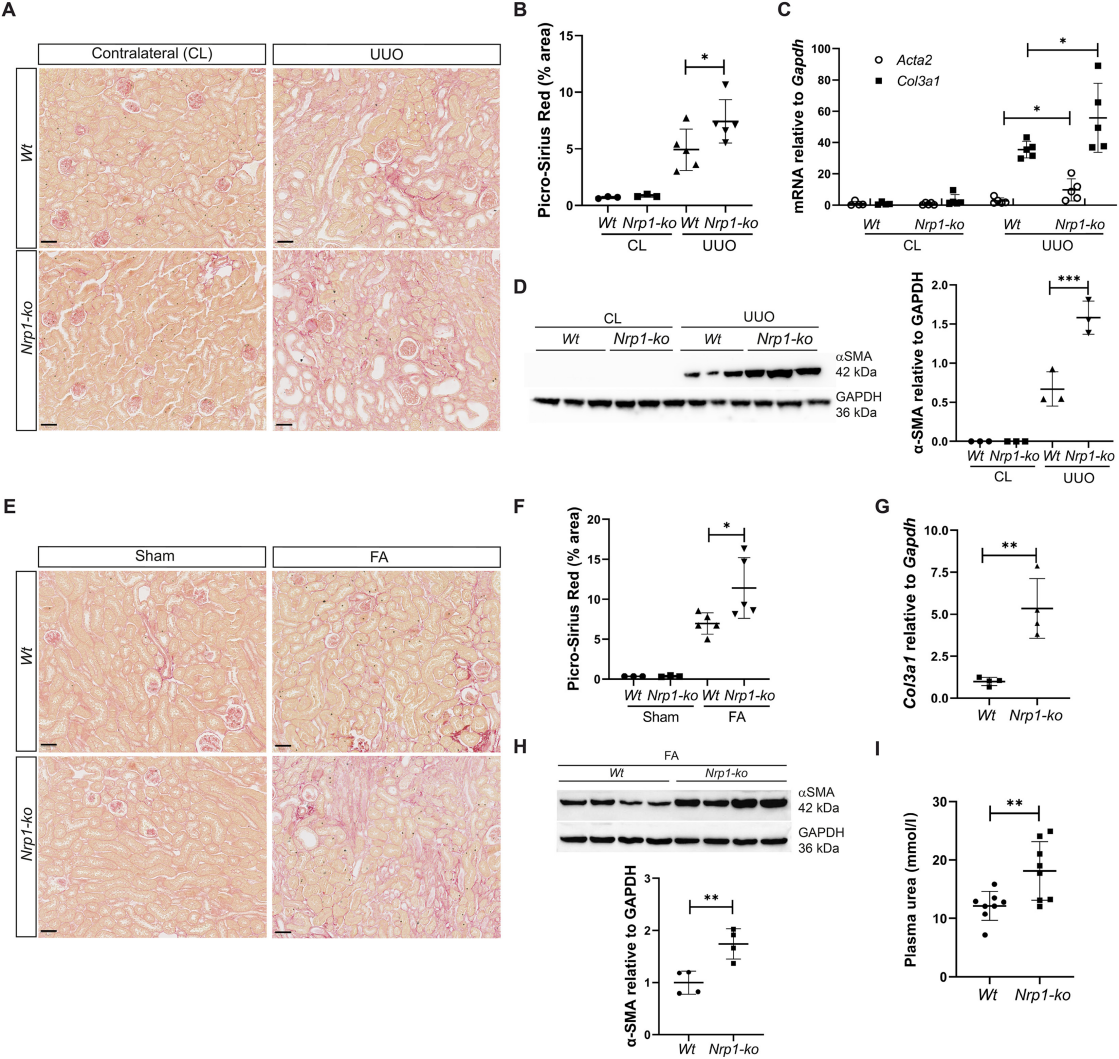
Lack of *Nrp1* promotes fibrogenesis in UUO and FA‐induced nephrotoxicity mouse models. (A–D) *Nrp1* deletion results in progressive fibrosis in UUO mouse model. UUO and contralateral (CL) kidneys of *WT* (*n* = 5) and *Nrp1‐ko* mice (*n* = 5) were (A) stained for Picrosirius red 10 days after surgery (scale bar, 50 μm) and (B) quantified. (C) Quantification of renal *Acta2* and *Col3a1* mRNA expression levels relative to *Gapdh* in UUO and CL kidneys of *Nrp1‐ko* (*n* = 5) and WT (*n* = 5) mice. (D) Western blotting of α‐SMA and GAPDH from CL and UUO kidneys derived from WT (*n* = 3) and *Nrp1‐ko* mice (*n* = 3) with corresponding quantification. (E–I) *Nrp1* deletion results in progressive fibrosis in FA‐induced nephrotoxicity model. (E) Picrosirius red staining of sham (*n* = 3) or FA‐treated (*n* = 5) renal tissues from *WT* and *Nrp1‐ko* animals 21 days after injection (scale bar, 50 μm) and (F) corresponding quantification. (G) Quantification of renal *Col3a1* mRNA expression levels relative to *Gapdh* in WT (*n* = 4) and *Nrp1‐ko* (*n* = 4) mice. (H) Western blotting of α‐SMA and GAPDH in WT (*n* = 4) and *Nrp1‐ko* (*n* = 4) mice treated with FA with corresponding quantification. (I) Plasma urea analysis of WT and *Nrp1‐ko* mice (*n* = 8) in FA‐treated animals 21 days after injection. *p < 0.05; **p < 0.01.

**Figure 2:**
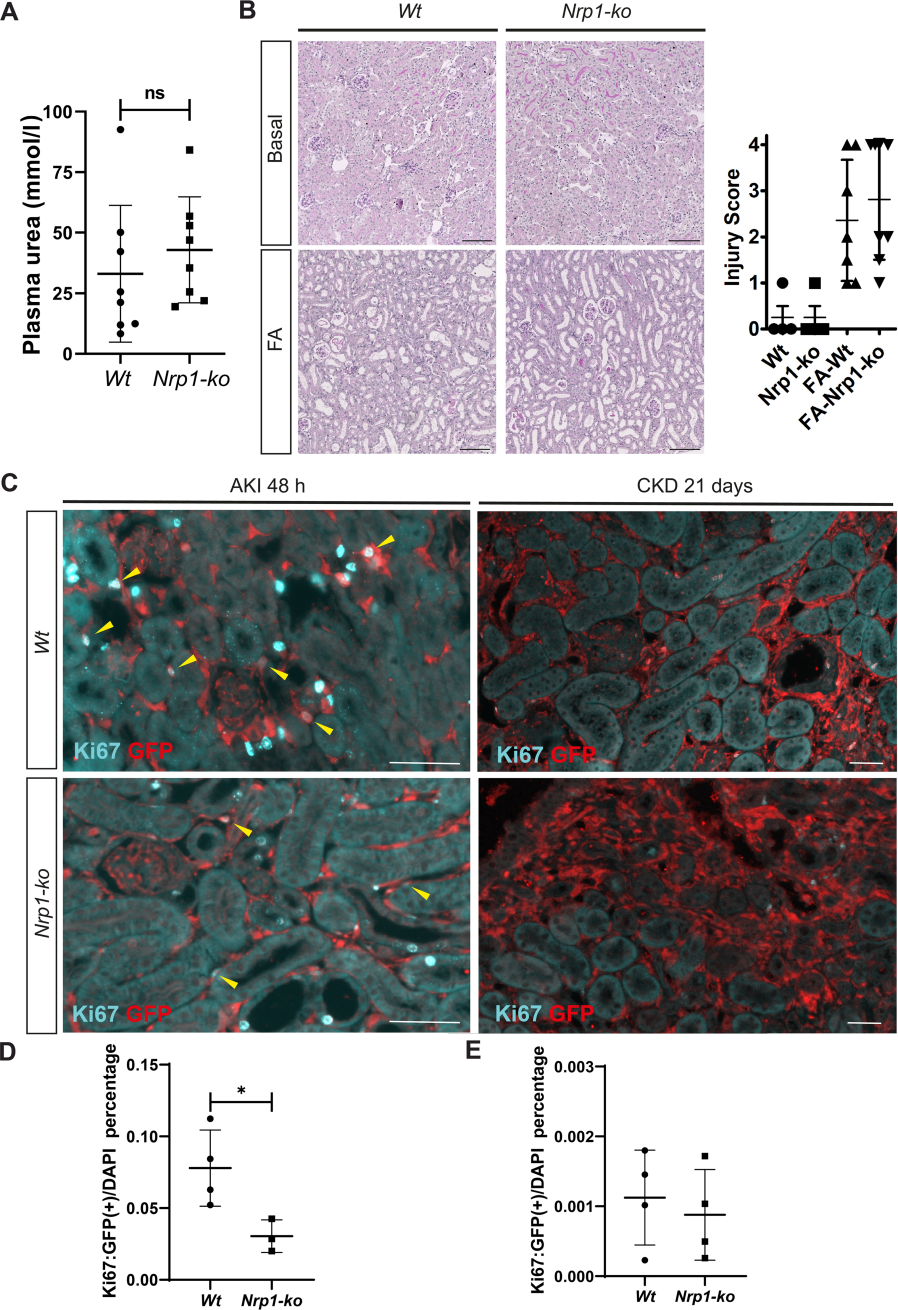
*Nrp1‐ko* fibroblasts displayed lower proliferation levels after AKI. (A) Plasma urea analysis of WT and *Nrp1‐ko* mice (*n* = 8) in FA‐treated animals 48 h after injection. (B) Periodic acid–Schiff (PAS) staining of WT and *Nrp1‐ko* kidneys under basal (*n* = 4) and FA‐treated 48 h after injection (*n* = 7), with corresponding quantification of injury score. Scale bar, 100 μm. (C) Immunofluorescence staining of GFP (red) and Ki67 (green) in WT and *Nrp1‐ko* kidneys 48 h and 21 days after FA injection. Scale bar, 100 μm. (D and E) Quantification of double Ki67‐ and GFP‐positive cells in WT (*n* = 4) and *Nrp1‐ko* (*n* = 3) mice at (D) 48 h and (E) 21 days after injection. Yellow arrowheads indicate Ki67/GFP double‐positive fibroblasts. ns: non‐significant; *p < 0.05.

**Figure 3:**
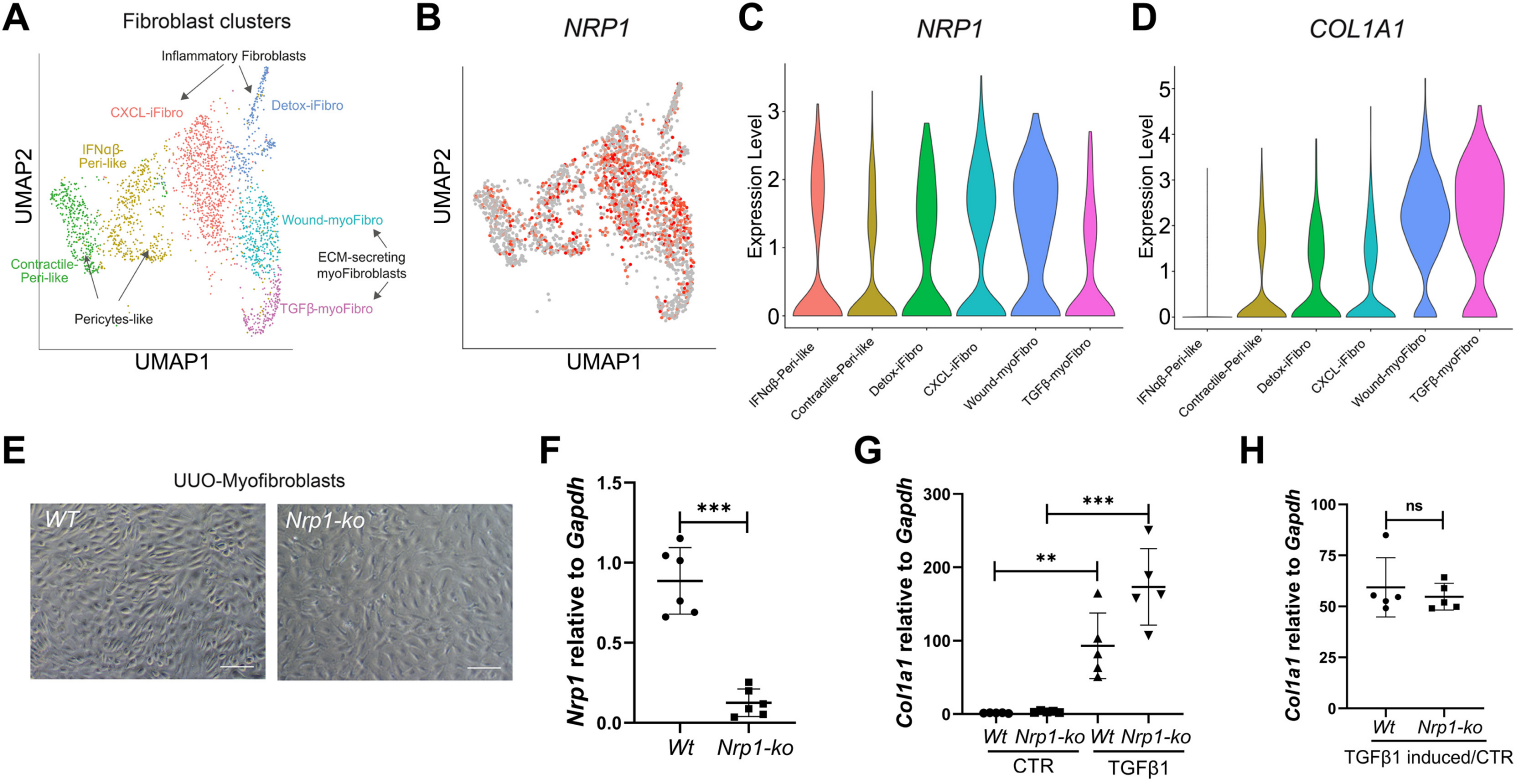
Identification of fibroblast cluster in CKD. (A) Uniform Manifold Approximation and Projection (UMAP) of scRNAseq data from 2,495 mesenchymal cells across 12 patients suffering or not from CKD, showing six clusters: IFNα/β‐Peri‐like; Contractile‐Peri‐like; detox‐iFibro; CXCL‐iFibro; Wound‐myoFibro, and TGFβ‐myoFibro. (B) UMAP and (C and D) violin plots showing *NRP1* and *COL1A1* expression across the six clusters. (E) WT and *Nrp1‐ko* myofibroblasts derived from UUO kidneys 10 days after surgery. (F) Quantification of *Nrp1* mRNA expression levels relative to *Gapdh* in *WT* (*n* = 3) and *Nrp1‐ko* (*n* = 3) myofibroblast cultures. Experiments were performed in duplicate. (G) Quantification of *Col1a1* mRNA expression relative to *Gapdh* in WT (*n* = 3) and *Nrp1‐ko* myofibroblast cultures (*n* = 3) under control (CTR) basal and TGFβ1‐induced conditions. Experiments were performed in duplicate. (H) *Col1a1* mRNA fold induction, calculated as amount of *Col1a1* mRNA produced upon TGFβ stimulation relative to CTR in TGFβ1‐induced WT and TGFβ1‐induced *Nrp1‐ko* myofibroblasts. ns: non‐significant; *p < 0.05; ***p < 0.001.

Previous work demonstrated that more than 94% of kidney myofibroblasts arose from *P0‐Cre* lineage‐labelled fibroblasts [3], providing us with a powerful genetic tool to specifically delete *Nrp1* in this cell population. We recently generated *P0‐Cre; Nrp1* ^*flox/flox*^; *Rosa* ^*YFP*^ (*Nrp1‐ko*) conditional mutants and confirmed NRP1 loss in kidney fibroblasts [13]. We further showed that constitutive deletion of *Nrp1* in this cell population did not affect basal renal structure and function [13]. In the present study, we challenged *Nrp1‐ko* mice with two well‐characterized models of kidney‐induced interstitial fibrosis: UUO and FA nephrotoxicity [3, 19] (Figure 1). Compared to *WT* mice subjected to UUO, *Nrp1‐ko* mice that received the same procedure had significantly increased deposition of renal ECM as assessed by Picrosirius red staining and collagen type III mRNA expression (Figure 1A–C). Furthermore, the mice showed a significantly more pronounced phenotypic switch from fibroblasts to myofibroblasts, as evidenced by increased α‐SMA protein and mRNA (*Acta2*) (Figure 1C,D). Likewise, subjecting *Nrp1‐ko* mice to FA‐induced nephrotoxicity significantly increased collagen and ECM production and myofibroblast transdifferentiation compared to FA‐treated WT mice (Figure 1E–H). Remarkably, 21 days after injection of FA, kidney function was more impaired in *Nrp1‐ko* mice than in WT mice (Figure 1I). This defect was not due to a differential response to AKI since both kidney function (Figure 2A) and kidney injury score (Figure 2B) were similar between *Nrp1‐ko* and WT mice. Overall, our results suggest an essential and protective role for NRP1 in resident fibroblasts after AKI. Since early proliferation of fibroblasts is required for kidney repair and regeneration after AKI [5], we measured the amount of double ki67‐, GFP‐positive fibroblasts in *Nrp1‐ko* and WT mice 48 h after injection of FA. *Nrp1‐ko* fibroblasts were observed to display lower proliferation levels than their WT counterparts (Figure 2C, left panel, Figure 2D), indicating that fibroblast functional recruitment was impaired in *Nrp1‐ko* mutants after AKI. Notably, very few ki67‐positive fibroblasts were detected in the CKD phase (Figure 2C, right panel, Figure 2E). We next used publicly available scRNAseq data of fibroblasts from patients with or without CKD [20]. Six clusters have already been described in this dataset, including pericytes (Contractile‐Peri‐like and IFNα/β‐Peri‐like), inflammatory fibroblasts (detox‐iFibro and CXCL‐iFibro), and ECM‐secreting myofibroblasts [Wound‐myoFibro and transforming growth factor beta (TGFβ)‐myoFibro] (Figure 3A). Interestingly, a differentiation process from fibroblasts/pericytes to TGFβ‐myoFibro has been suggested, with Wound‐myoFibro being the final state before TGFβ‐myoFibro. Differentially expressed gene analysis between these clusters revealed an upregulation of the wound healing processes in Wound‐myoFibro, and a response to TGFβ and matrix remodelling in TGFβ‐myoFibro [20]. We found that the vast majority of NRP1‐positive human myofibroblasts derived from the inflammatory CXCL_iFibro and Wound‐myoFibro clusters rather than the TGFβ‐myoFibro cluster (Figure 3B,C). Interestingly, CXCL_iFibro were not directly involved in ECM secretion (Figure 3D), and the switch from CXL_iFibro to Wound‐myoFibro to TGFβ‐myoFibro is essential in ECM secretion [20]. These findings suggest that Nrp1‐positive fibroblasts are not entirely implicated in TGFβ‐induced ECM production but instead contribute to inflammation and wound‐healing regulation following kidney injury.

To further validate these findings, we cultured myofibroblasts extracted from UUO‐derived *Nrp1*‐*ko* kidneys and UUO‐derived WT kidneys, as previously described [21] (Figure 3E). As expected, *Nrp1* transcripts were specifically downregulated in *Nrp1‐ko* myofibroblasts (Figure 3F). Since NRP1 has been shown to increase TGFβ‐dependent collagen production in a model of liver fibrosis [10], we examined the response of *Nrp1‐ko* myofibroblasts to 24 h TGFβ stimulation (Figure 3G). Type I collagen synthesis increased significantly in both *Nrp1‐ko* and WT myofibroblasts (Figure 3G), with comparable fold induction (52.85‐fold in WT *versus* 50.87‐fold in *Nrp1‐ko*; Figure 3H). These results suggest that *Nrp1* deletion does not impair TGFβ signalling but rather controls fibroblast lineage differentiation.

To validate that NRP1 signalling is essential to maintain fibroblast proliferation and prevent transdifferentiation into myofibroblasts, we quantified the amount of *P0‐Cre*‐positive fibroblasts which had differentiated into α‐SMA myofibroblasts in the FA‐induced nephrotoxicity and UUO models. We found a higher number of double‐labelled α‐SMA/GFP cells in *Nrp1‐ko* than in WT mice, suggesting that *Nrp1* deletion enhanced myofibroblast content from the progenitor pool population (supplementary material, Figure S2).

**Figure 4:**
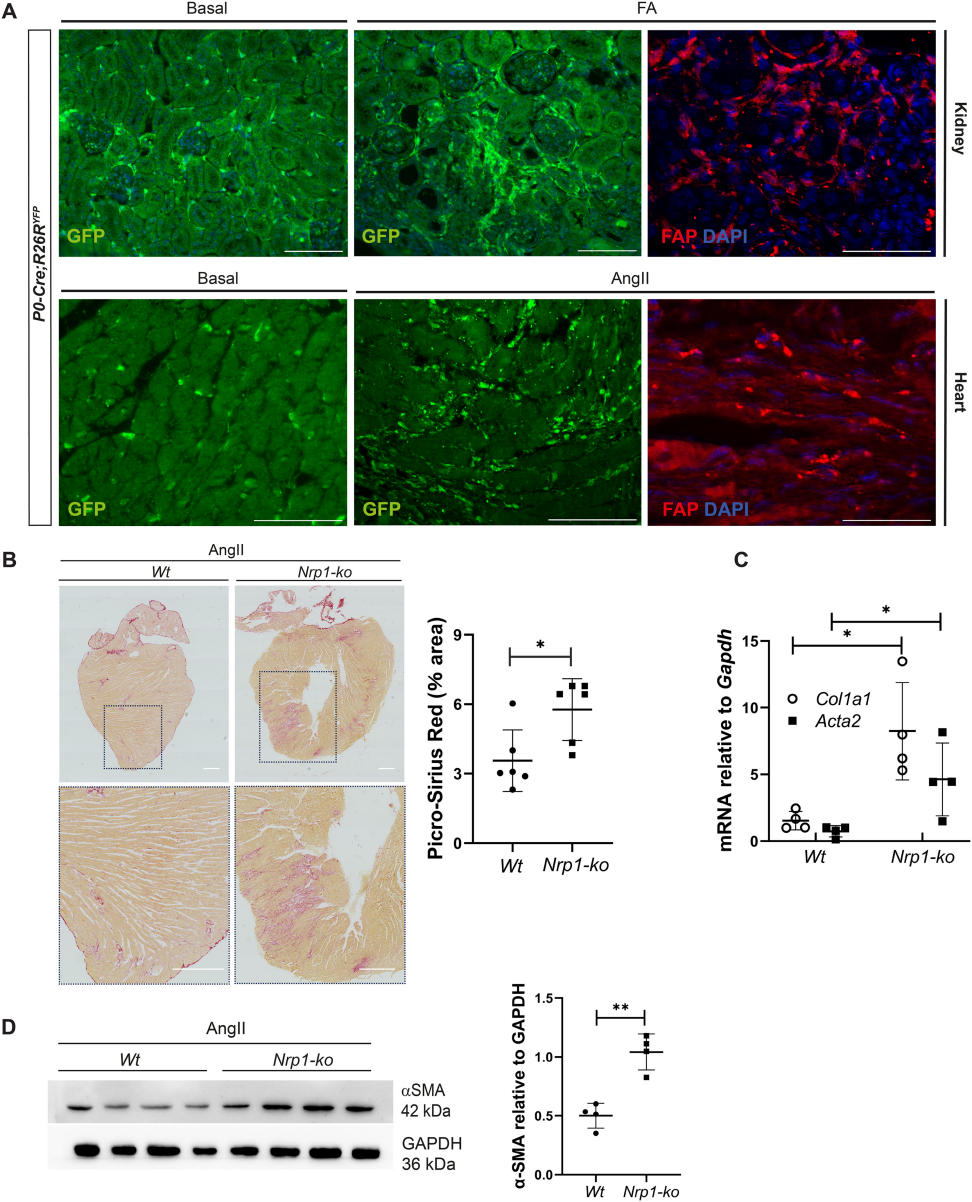
Lack of *Nrp1* promotes cardiac fibrogenesis in AngII‐induced fibrosis mouse model. (A) *P0‐Cre;R26R* ^*YFP*^ mice were immunolabelled for GFP in basal kidneys or FA‐treated kidneys (21 days, upper panel) and in basal hearts or AngII‐induced hearts (28 days, lower panels). Immunostaining for FAP (red) was performed on fibrotic kidneys and fibrotic hearts (upper and lower right panels). Scale bar, 50 μm. (B) Picrosirius red staining of AngII‐induced hearts of WT and Nrp1‐ko mice, with boxed areas shown at higher magnification, and corresponding quantification (*n* = 6). (C) Quantification of cardiac *Col1a1* and *Acta2* mRNA expression levels relative to *Gapdh* in WT and *Nrp1‐ko* mice treated with AngII for 28 days (*n* = 4). (D) Western blotting analysis of cardiac tissue for α‐SMA and GAPDH in WT and *Nrp1‐ko* mice treated with AngII for 28 days (*n* = 4) and corresponding quantification. *p < 0.05; **p < 0.01.

As *P0‐Cre* lineage‐labelled progenitors also target cardiac tissue [22], we next performed lineage tracing experiments using *P0‐Cre; Rosa* ^*YFP*^ mice and collected kidneys and hearts under both basal and fibrotic conditions (Figure 4). We found that renal and cardiac fibroblasts/myofibroblasts derived from the same GFP‐positive progenitor cell population (Figure 4A, left and middle panels, respectively). Both renal and cardiac fibroblasts expressed the fibroblast activation protein marker FAP (Figure 4A, right panels) [23]. We next challenged *Nrp1‐ko* mice with a model of AngII‐induced interstitial fibrosis using osmotic pump delivery for 28 days [23]. While deletion of *Nrp1* in *P0‐Cre* lineage‐labelled progenitors did not alter heart structure under basal conditions (supplementary material, Figure S1), hearts of *Nrp1‐ko* treated with AngII minipumps had significantly increased deposition of ECM as assessed by quantification of Picrosirius red staining and mRNA expression of type I collagen (*Col1a1*) and *Acta2* I (Figure 4B,C). Furthermore, *Nrp1‐ko* mice had significantly more α‐SMA‐positive cardiac myofibroblasts than their WT counterparts (Figure 4D).

## Discussion

In this study, we specifically deleted *Nrp1* in kidney resident fibroblasts during fibrosis progression. We found that NRP1 signalling is essential for maintaining fibroblast proliferation and preventing their transdifferentiation into myofibroblasts. *Nrp1‐ko* mice, subjected to two models of kidney‐induced interstitial fibrosis displayed exacerbated ECM deposition and an increased number of myofibroblasts. Because NRP1 is able to enhance TGFβ signalling during liver fibrogenesis [10], we used *ex vivo* myofibroblast cultures combined with human scRNAseq data to assess whether this mechanism also contributed to CKD progression. We found that NRP1 is predominantly expressed in a subpopulation of fibroblasts involved in wound/healing, with a limited role in ECM deposition through TGFβ signalling. A recent study also suggested that targeting tubular NRP1 signalling may represent a promising strategy for the treatment of AKI and subsequent CKD [11]. In contrast, our cell‐specific analysis, integrating both mouse genetics and human data, suggests a protective role of fibroblast‐derived NRP1 in kidney fibrosis progression. These opposing results emphasise the complexity of NRP1 signalling in both physiological [12, 13, 14] and pathophysiological contexts, as well as the essential role played by cell‐type‐specific compartment in experimental outcomes. Two studies by Kuppe *et al* identified PDGFR‐β‐positive fibroblasts as major matrix‐producing cells both in the kidney and heart tissue [4, 24]. Our lineage‐tracing experiments extend these findings by demonstrating that renal and cardiac fibroblasts originate from the same progenitor cells. In addition, deletion of *Nrp1* in this *P0‐Cre* lineage‐labelled progenitors affected both the heart and kidney to a similar degree when subjected to experimental models of fibrosis.

These findings suggest that the heart and kidney share common pathways to organ fibrosis, which may be used as therapeutic targets in both organs. Furthermore, CKD‐driven cardiac fibrosis is a major contributor to heart failure and increased cardiovascular mortality. From a clinical perspective, therapeutic strategies aimed at reducing fibrosis in both organs would be relevant. For instance, the use of chimeric antigen receptor (CAR)‐T cells targeting FAP has proven effective at reducing fibrosis and restoring cardiac function [23]. Since FAP upregulation is also observed in kidney myofibroblasts, further studies are warranted to determine whether this strategy could be beneficial in mitigating kidney fibrosis.

## Author contributions statement

YS, SP, LL, PF, SF, CaC and AC conducted the experiments and acquired the data. YS, CaC, ChC and AC analysed the data. CEC and HF provided reagents. AC and ChC designed the research study. YS, CaC, ChC and AC wrote the manuscript with contributions from all the authors.

## Supporting information


**Supplementary materials** **and methods**
**Figure S1**. Basal heart structure is maintained in *Nrp1‐ko* mice
**Figure S2**. *Nrp1* prevents fibroblast differentiation into myofibroblasts

## Data Availability

The data that support the findings of this study are available from the corresponding authors upon reasonable request.
