# Supplementary material for: Fibroblast‐derived neuropilin 1 alleviates renal fibrosis progression
Source: J Pathol. 2025 Dec 23;268(2):127–34. doi: 10.1002/path.70009 (PMC12805606; doi:10.1002/path.70009)
Supplement: Supplementary file 1 — Supplementary materials and methods Figure S1. Basal heart structure is maintained in Nrp1‐ko mice Figure S2. Nrp1 prevents fibroblast differentiation into myofibroblasts [file PATH-268-127-s001.docx]

**Fibroblast-derived neuropilin 1 alleviates renal fibrosis progression**

Y Shen *et al. J Pathol* <https://doi.org/10.1002/path.70009>

**Supplementary materials and methods**

**Supplementary Figures S1 and S2**

Reference numbers refer to the main text list

**Supplementary materials and methods**

### **Western blotting**

Proteins from kidney or heart tissue were extracted using RIPA lysis buffer supplemented with a cocktail of protease inhibitors. Total protein concentration was measured using a Bradford assay, and equal amounts of proteins (up to 50 µg) were loaded onto a NuPAGE 4–12% gradient gel (Invitrogen, Waltham, MA, USA) and transferred to a PVDF membrane (BioRad, Hercules, CA, USA). Primary antibodies were rabbit anti-α-SMA (ab5694, Abcam, Cambridge, UK) and rabbit anti-GAPDH (Sigma-Aldrich, St Louis, MO, USA). Blots were developed using ECL advance chemiluminescent reagent (Bio-Rad) and imaged using a ChemiDoc MP Imaging System (Bio-Rad). Data were expressed as mean ± SD of optical density (OD) values.

### **RNA extraction and real-time analysis**

Total RNA from kidney or heart tissue was extracted using TRIReagent (Molecular Research Center, Cincinnati, OH, USA). cDNA was synthesised from 1 µg of total RNA using the Maxima First Strand cDNA Synthesis Kit (Thermo Fisher Scientific, Waltham, MA, USA). Real-time PCR was performed with the Roche Light Cycler 480 detection system using SYBR green PCR master Mix (Roche Diagnostics, Rotkreuz, Zug, Switzerland). Specific primers for target mRNAs: *Gapdh* 5’-TGCGACTTCAACAGCAACTC-3’ and 5’-CTTGCTCAGTGTCCTTGCTG-3’, *Nrp1* 5’-CCATTATAGACAGCACCATCC-3’ and 5’-AAGTTGCCATCTCCTGTATG-3′, *Col3a1* 5’-TCCCCTGGAATCTGTGAATC-3’ and 5’- TGAGTCGAATTGGGGAGAAT-3’, *Col1a1* 5’- CATGTTCAGCTTTGTGGACCT-3’ and 5’-GCAGCTGACTTCAGGGATGT-3’, *Acta2* 5’-TCAGCGCCTCCAGTTCCT-3’ and 5’-AAAAAAAACCACGAGTAACAAATCAA-3’ were used for amplification and a standard curve generated for each targeted transcript. The unknown relative values were extrapolated from the standard curve and expression levels normalised to *Gapdh* expression. The average of triplicate reactions was used as the value for the sample.

### **Genotyping primers**

The following genotyping primers were used:

*Nrp1 Flox Forw* 5’-AAGGAGTGGCACAGCATCTT-3’

*Nrp1 Flox Rev* 5’-TCACACCCAAACTTCCTTCC-3’

*Cre Forw* 5’-TGGAAAATGCTTCTGTCCGTTTGC-3’

*Cre Rev* 5’-AACGAACCTGGTCGAAATCAGTG-3’

### **Myofibroblast *ex vivo* explants**

Primary myofibroblasts were obtained from UUO kidneys of WT (*n* = 3) and *Nrp1-ko* mice (*n* = 3). The obstructed kidneys were removed 10 days following UUO, sectioned into 1-mm^3^ tissue fragments, placed in a Petri dish, and immersed in DMEM supplemented with 10% FBS. Cells were cultured at 37 °C in a humidified incubator with 5% CO2 and utilised between passages 5 and 12. Myofibroblast stimulation was performed by adding 5 ng/ml of TGFβ (240-B, R&D Systems, Minneapolis, MN, USA) to 1% FBS DMEM medium for 24 h, as previously described [21]. Reference number refers to the main text list.


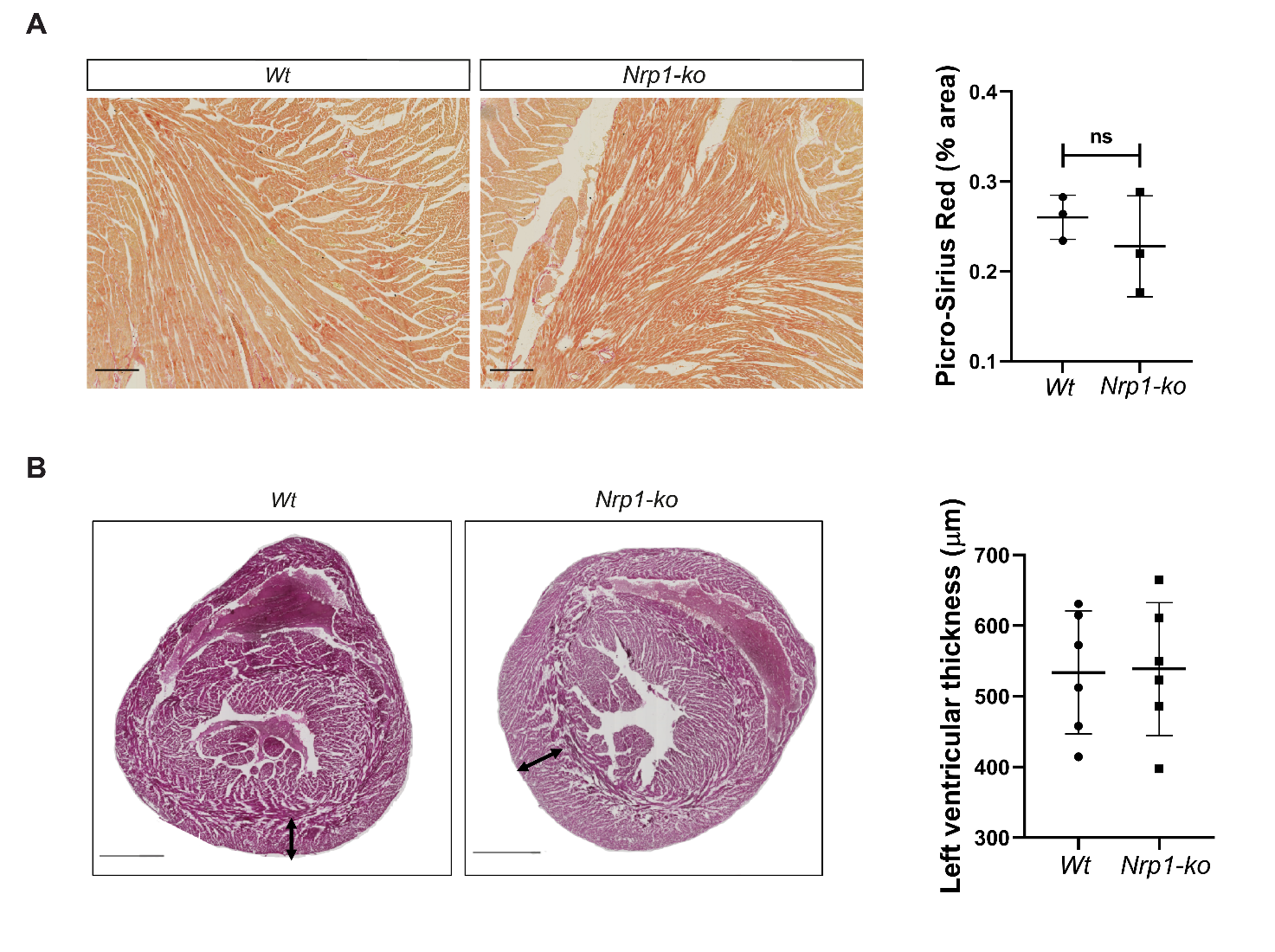


**Figure S1. Basal heart structure is maintained in *Nrp1-ko* mice.** (A) Picrosirius red staining of WT (*n* = 3) and *Nrp1-ko* (*n* = 3) hearts under basal conditions, with corresponding quantification. Scale bars, 50 µm. ns, non-significant. (B) H&E staining of WT (*n* = 6) and *Nrp1-ko* (*n* = 6) hearts under basal conditions, with measurements of left ventricular thickness and quantification. Black double arrow indicates left ventricular thickness. Scale bars, 1 mm.

**
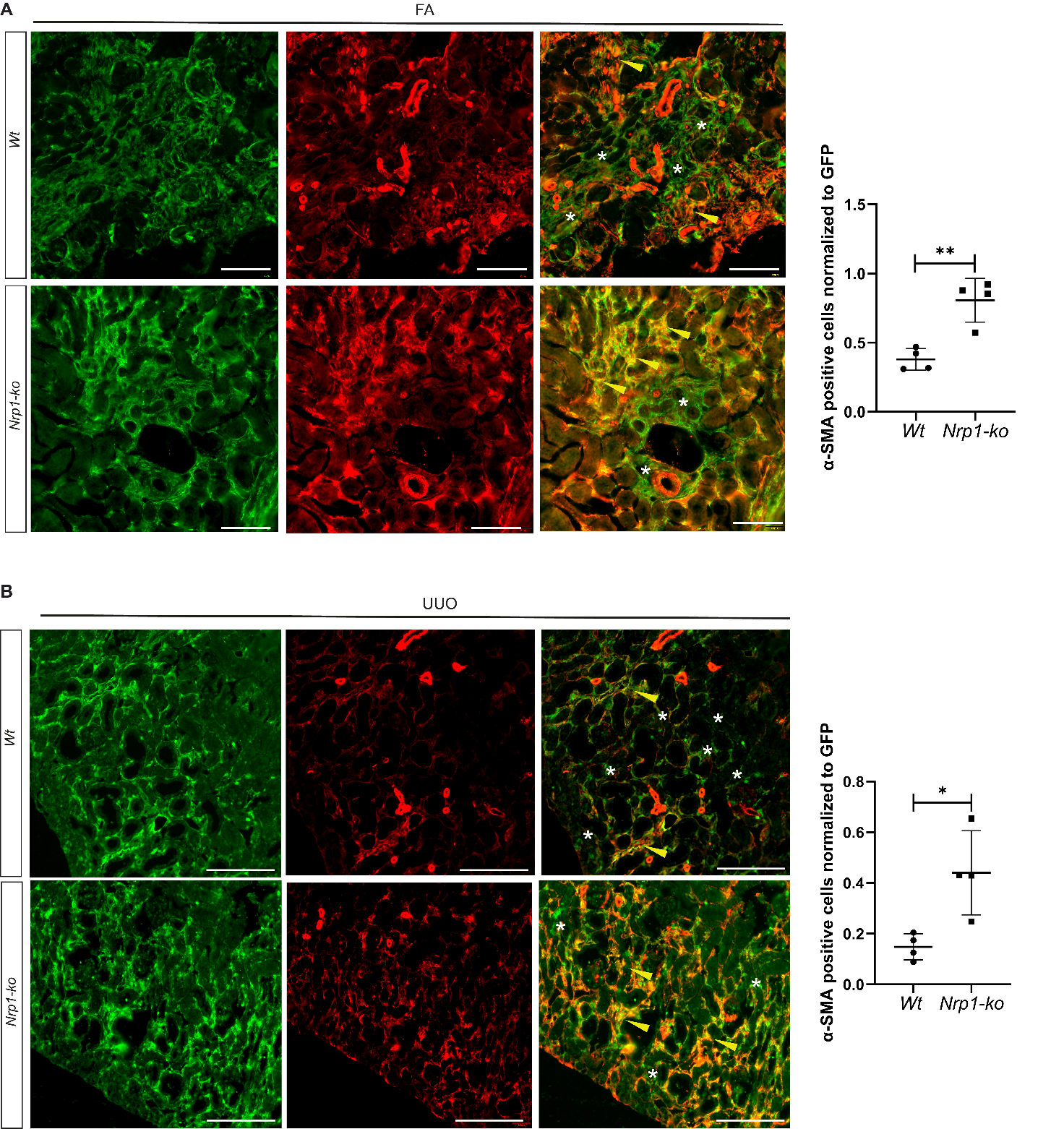
**

**Figure S2. *Nrp1* prevents fibroblast differentiation into myofibroblasts.** Immunofluorescent staining of α-SMA (red) and GFP (green) in WT and *Nrp1-ko* kidneys following treatment with (A) FA 21 days after injection or (B) UUO 10 days after surgery. Yellow arrowheads indicate α-SMA/GFP double-positive myofibroblasts, and white asterisks indicate undifferentiated *P0-cre* positive fibroblasts. Scale bars, 100 µm **p* < 0.05; ***p* < 0.01.
